# Preliminary Study on Species Diversity and Community Characteristics of Gamasid Mites on Small Mammals in Three Parallel Rivers Area of China

**DOI:** 10.3390/ani12223217

**Published:** 2022-11-20

**Authors:** Juan-Xiu Zhou, Xian-Guo Guo, Wen-Yu Song, Cheng-Fu Zhao, Zhi-Wei Zhang, Rong Fan, Ting Chen, Yan Lv, Peng-Wu Yin, Dao-Chao Jin

**Affiliations:** 1 Institute of Pathogens and Vectors, Yunnan Provincial Key Laboratory for Zoonosis Control and Prevention, Dali University, Dali 671000, China; 2 Institute of Entomology, Guizhou University, Guiyang 550025, China

**Keywords:** acari, gamasid mites, ectoparasites, species diversity, community, Three Parallel Rivers Area, China

## Abstract

**Simple Summary:**

In addition to causing dermatitis, some gamasid mites are also the vector or potential vector of some zoonotic diseases. Located in the northwest Yunnan Province of southwest China, the Three Parallel Rivers Area of China is one of the hotspots of biodiversity research in the world. Based on the previous field survey in the Three Parallel Rivers Area from 2001 to 2015, this paper reports the species diversity and basic community characteristics of gamasid mites on small mammals in this unique geographical area for the first time. From the body surface of 3830 small mammal hosts, 26,048 gamasid mites were collected and identified as 10 families, 21 genera and 82 species (excluding 847 unidentified specimens) with high species diversity. The species diversity of the gamasid mite community fluctuated greatly in different elevation gradients. The highest peaks of species richness and *β* diversity appeared at altitudes of 3000–3500 m (*S* = 42) and 1500–2000 m (*β* = 17.5), respectively. The species abundance distribution of the mites was successfully fitted by Preston’s lognormal model, and the total number of gamasid mite species in the Three Parallel Rivers Area was estimated to be 153 species.

**Abstract:**

(1) Background: Gamasid mites are a large group of arthropods, and some of them are of medical importance. Besides directly biting humans and causing dermatitis, some gamasid mites are the vector of rickettsialpox and potential vector of hemorrhagic fever with renal syndrome (HFRS). The Three Parallel Rivers Area of China is one of the hotspots of biodiversity research in the world, with complicated topographic landforms, different types of vegetation, special elevation gradients and high biodiversity. (2) Methods: Species richness (*S*): the Shannon–Wiener diversity index (*H*), Simpson dominance index (*D*) and Pielou evenness index (*E*) were used to analyze the basic community structure. The *β* diversity (Cody index) was used to reflect the diversity difference between any two adjacent elevation gradients. The method based on Preston’s lognormal model for species abundance distribution was used to estimate the total number of gamasid mite species. (3) Results: A total of 3830 small mammal hosts captured from the nine survey sites were identified as 44 species, 27 genera and nine families in five orders. *Apodemus chevrieri*, *Eothenomys miletus* and *A. draco* were the dominant host species with a total constituent ratio *C_r_* = 52.037%. From the body surface of the hosts, 26,048 gamasid mites were collected and identified as 10 families, 21 genera and 82 species (excluding 847 unidentified specimens) with high species richness (*S* = 82) and diversity (*H* = 2.33). The three dominant mite species were *Dipolaelaps anourosorecis*, *Laelaps nuttalli* and *L. echidninus*, with a total *C_r_* = 64.46% (16,791/26,048). There are significant differences in the species composition, species diversity and dominant species of gamasid mites on different hosts. The species diversity of the mite community fluctuated greatly in different elevation gradients. The highest peaks of species richness and *β* diversity appeared at altitudes of 3000–3500 m (*S* = 42) and 1500–2000 m (*β* = 17.5), respectively. The species abundance distribution of the mites was successfully fitted by Preston’s lognormal model with $\hat{S}\left(R\right)=19e^{-{\left[0.22\left(R-0\right)\right]}^{2}}$ (*α* = 0.22, *R*^2^ = 0.9879). Based on fitting the theoretical curve by Preston’s model, the total number of gamasid mite species was estimated to be 153 species. (4) Conclusions: Gamasid mites on small mammals are abundant with complex community structures and high species diversity in the Three Parallel Rivers Area of China. There is an apparent community heterogeneity of the mites on different hosts and in different environments.

## 1. Introduction

Gamasid mites are a large group of arthropods with complex and diverse ecological behaviors. It is estimated that about 8280 species of gamasid mites have been recorded worldwide, with more than 600 species documented in China [1,2]. Gamasid mites belong to the suborder Mesostigmata and order Parasitiformes in the subclass Acari of the class Arachnoidea [3,4]. There are five primary stages in the life cycle of gamasid mites, the egg, larva, protonymph (first nymph), deutonymph (second nymph) and adult (the male and female). The majority of gamasid mites are soil-living and free-living with complex habitats and ecological behaviors. For ectoparasitic gamasid mites, nearly all stages (except the egg) can be found on the body surface of small mammals and other animal hosts which are used as food sources of the mites [5,6,7]. Some species of ectoparasitic gamasid mites can transmit some zoonoses, and some endoparasitic gamasid mites can directly parasitize the lungs of animals or people, causing pulmonary acariasis [8,9,10]. As a whole group of arthropods, ectoparasitic gamasid mites (including facultatively and occasionally ectoparasitic species) have extensive hosts, including small mammals, aves (birds), reptiles, amphibians and other arthropods. Besides directly stinging the human skin and causing dermatitis, some species of gamasid mites are suspected to be associated with the transmission of more than 20 zoonotic diseases, with some species being the transmitting vector of rickettsialpox and potential vectors of hemorrhagic fever with renal syndrome (HFRS) [11,12,13,14,15,16]. Some gamasid mites can be the intermediate host of cotton rat filarial (*Litomosoides carinii* Travassos, 1919), the pathogen of cotton rat filarial disease, causing harm to experimental rats and mice [17,18].

Located in the Longitudinal Range-Gorge Region of the Hengduan Mountains in the eastern Himalayas, the Three Parallel Rivers Area is a unique geographical region in the northwest of Yunnan Province, southwest China. It is a transitional zone between the Tibetan Plateau (Qinghai–Tibet Plateau) and Yunnan–Guizhou Plateau [19,20,21,22]. As the intersection of the three geographical regions of East Asia, South Asia and the Tibetan Plateau, the Three Parallel Rivers Area borders Myanmar to the west, Tibet Autonomous Region to the northwest and Sichuan Province to the northeast and east [23,24]. In the northwest of Yunnan Province, there are three rivers (Jinshajiang, Lancangjiang and Nujiang rivers) originating from the Tibetan Plateau, flowing from the north towards the south [19,24]. The three rivers parallel each other, passing through Gaoligong Mountain, Nushan Mountain, Yunling Mountain and Shaluli Mountain without intersecting [23,25]. This area has a series of deep valleys, and four central high mountains are divided by three rivers, forming a very complex longitudinal mountain–valley landscape [24,25]. The topography, landscape, ecological environment and climate type in the area are incredibly complex with considerable changes in altitudes ranging from 720 m to 6740 m, which may contribute to the complex composition and high species diversity of animals and plants. It is one of the areas with the world’s most diverse species and has become one of the hotspots in biodiversity research [26,27]. Ma et al. conducted a particular study on plant species diversity in the Three Parallel Rivers Area and found 98 species of plants [22], Renee Mullen et al. conducted a regional-level assessment of the alpine flora and reported 369 species of plants [28].

Rodents and some other small mammals are not only the important infectious source of some zoonoses (zoonotic diseases), but are also the most important hosts of ectoparasitic gamasid mites [2,4,29], and therefore it is of significance to study the mites on their body surface. Some previous studies have shown that the Three Parallel Rivers Area and its adjacent areas are rich in species of aves and mammals (including small mammals), but unfortunately few studies have dealt with ectoparasitic mites (including ectoparasitic gamasid mites) in the areas [30,31,32,33]. According to the field investigation data of nine survey sites in the Three Parallel Rivers Area from 2001 to 2015, this paper conducted a preliminary study on the species diversity and basic community characteristics of gamasid mites on small mammals in this unique area for the first time.

## 2. Materials and Methods

### 2.1. Field Survey Sites

**Figure 1:**
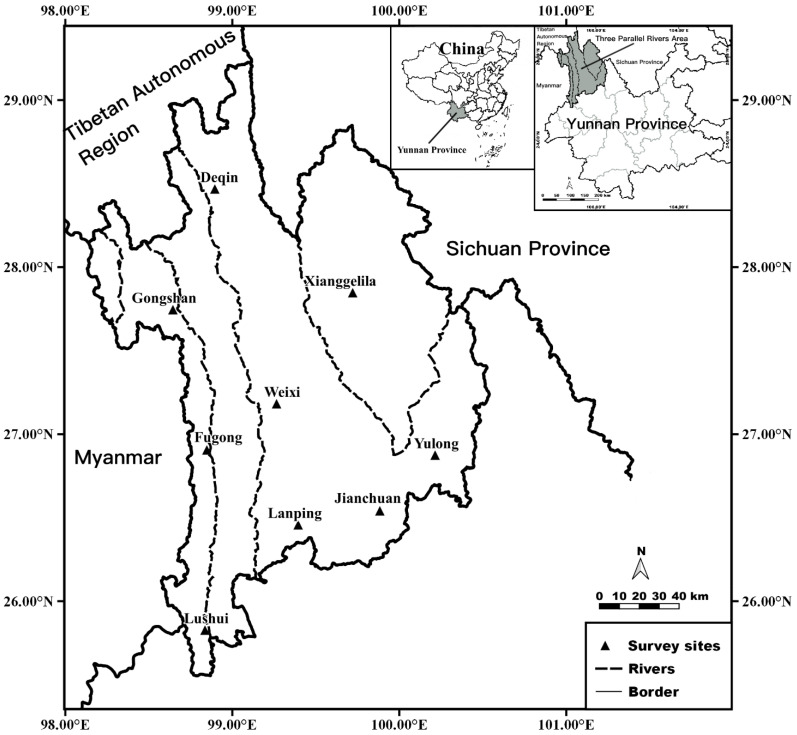
Geographical location of Three Parallel Rivers Area and nine survey sites in northwest Yunnan of China (2001–2015).

The original data came from a long-term field investigation of nine survey sites in the Three Parallel Rivers Area of northwest Yunnan Province, southwest China between 2001 and 2015. The nine survey sites are as follows: Gongshan (27°44′40″ N, 98°39′55″ E), Jianchuan (26°32′26″ N, 99°54′16″ E), Yulong (26°52′27″ N, 100°14′08″ E), Xianggelila (27°50′45″ N, 99°44′31″ E), Lanping (26°27′24″ N, 99°24′55″ E), Weixi (27°10′50″ N, 99°17′09″ E), Deqin (28°28’01” N, 98°54’59” E), Lushui (25°49′34″ N, 98°51′27″ E) and Fugong (26°54′19″ N, 98°52′07″ E) (Figure 1 in Results).

### 2.2. Gamasid Mites Collection and Identification

In the involved nine survey sites, mousetraps were placed randomly in different habitats (residential areas, cultivated farmlands, grasslands, shrubs, forests, etc.) in the evening and checked the following morning. The trapped rodents and other small mammals (hosts) were separately placed in marked white cloth bags and then transported to the field laboratory where gamasid mites were collected. The captured hosts were anesthetized with ether and then gamasid mites on their body surface were collected one by one. According to the appearance (body sizes, shape and coat color), various comprehensive measurements (body length, body weight, tail length, ear height and hind foot length, etc.) and other morphological features, each host was identified into species [34,35,36]. The collected gamasid mites from each host were preserved in a vial containing 70% ethanol and then mounted onto a glass slide in Hoyer’s mounting medium. The mounted mites were finally identified into species under a microscope based on related taxonomic literature and identification books which contain a series of identification keys [4,37,38,39]. The use of animals (including animal euthanasia) for our research was officially approved by the Animals’ Ethics Committee of Dali University, under permission number DLDXLL2020-1104. Representative specimens of animal hosts and gamasid mites were deposited in the specimen repository of the Institute of Pathogens and Vectors, Dali University, Dali, Yunnan, China.

### 2.3. Species Diversity and Basic Community Structure Statistics

Together with species richness (*S*), which was used to reflect species diversity, the Shannon–Wiener diversity index (*H*), Simpson dominance index (*D*) and Pielou evenness index (*E*) were used to analyze the basic community structure of the small mammal hosts and gamasid mites [40,41]. The *β* diversity (Cody index) was used to reflect the diversity difference between any two adjacent elevation gradients [42,43].

(1) $$H=-\sum_{i=1}^{S}P_{i}lnP_{i}$$

(2) $$D=\sum_{i=1}^{S}P_{i}^{2}$$

(3) $$E=\frac{H}{lnS}$$

(4) $$\beta =\frac{g\left(h\right)+l\left(h\right)}{2}$$

In the above formulas, *S* represents species richness (the number of species), *P_i_* represents the proportion of the individuals of gamasid mite species *i* (or hosts species *i*) to the total number of all the species, *g*(*h*) is the number of species increased along the elevation gradient *h* (the number of species that were absent in the previous gradient and newly emerged in the following gradient), *l*(*h*) is the number of species decreased along the elevation gradient *h* (the number of species present in the previous gradient but absent in the following gradient).

### 2.4. Species Abundance Distribution and Total Species Estimation

All gamasid mites from nine survey sites in the Three Parallel Rivers Area were taken as a gamasid mite community unit. To illustrate the species abundance distribution of the gamasid mite community on small mammals, a semi-logarithmic rectangular system was established. The *X*-axis was labeled with log intervals based on log_3_M, which indicates the individuals of gamasid mites, and the *Y*-axis was marked with arithmetic scales, representing the number of gamasid mite species. Based on the following formulae, Preston’s lognormal model was used to fit the theoretical curve of species abundance distribution, and the fitting goodness (*R*^2^) was calculated. According to the final fitting goodness (*R*^2^), the species abundance theoretical curve equation was established and the theoretical curve was drawn [44,45]. The total number of gamasid mite species (*S_T_*) was then estimated. According to the total number of species (*S_T_*), the likely missed number of species (*S_M_*) in the field survey was approximately calculated [46,47].

(5) $$S\left(R\right)=S_{0}e^{-{\left[a\left(R-R_{0}\right)\right]}^{2}}\;(e=2.71828\;\ldots )\;(\text{Preston’s}\;\text{lognormal}\;\text{model})$$

(6) $$R^{2}=1-\sum_{R=0}^{m}{\left[S^{\prime }\left(R\right)-S\left(R\right)\right]}^{2}/\sum_{R=0}^{m}{\left[S^{\prime }\left(R\right)-\bar{S}\left(R\right)\right]}^{2}$$

(7) $$\bar{S}\left(R\right)=\frac{1}{m}\sum_{R=0}^{m}S^{\prime }\left(R\right)$$

(8) $$S_{T}=\frac{S_{0}\sqrt{\pi }}{\alpha }$$

(9) $$S_{M}=S_{T}-S_{A}$$

In the above formulas, *S*(*R*) represents the theoretical number of gamasid species at the *R*-th log interval, *S*_0_ represents the number of gamasid mite species at the *R*_0_ log interval, *m* is the number of log intervals and *R*_0_ represents the mode log interval. The value of *α* was determined according to the best fitting goodness, *R*^2^. *S*’(*R*) represents the actual number of gamasid mite species at *R*-th log interval and $\bar{S}\left(R\right)$ represents the average number of gamasid mite species for each log interval. *S_T_* is the total theoretical number of gamasid mite species in the community (the total expected number of gamasid mite species), *S_M_* is the number of gamasid mite species likely missed in the field survey and *S_A_* is the number of gamasid mite species actually collected in the field survey.

## 3. Results

### 3.1. Classification and Identification of Gamasid Mites and Their Small Mammal Hosts

Between 2001 and 2015, a total of 26,895 individuals of gamasid mites were collected from the body surface of 3830 small mammal hosts from nine survey sites in the Three Parallel Rivers Area of China (Figure 1). The 3830 small mammal hosts were identified as 44 species, 27 genera and nine families in five orders: Rodentia, Eulipotyphla (previously Insectivora), Scandentia, Lagomorpha and Carnivora (small carnivores only) (Table 1). Of the captured small mammal hosts, rodents (order Rodentia) accounted for 75.00% of species (33/44) and 91.83% of individuals (3517/3830), which are the most dominant category of small mammal hosts in the Three Parallel Rivers Area, and the order Eulipotyphla came next (7.57%, 290/3830). The order Carnivora was the least, accounting for only 0.03% (1/3830) (Table 1). The total proportion of non-rodent small mammal hosts was 8.17% (313/3830). Of 33 species and 3517 individuals of rodent hosts, the species and individuals of murid rodents in the family Muridae accounted for 43.18% (19/44) and 73.55% (2817/3830), and the ratio of murid versus non-murid rodents was 4.02 (2817/700). The survey sites and collected individuals of 44 captured small mammal host species were listed in “Appendix A: Taxonomic checklist of small mammals captured from nine survey sites in Three Parallel Rivers Area of northwest Yunnan, China (2001–2015)”.

Of the 26,895 collected gamasid mites, 26,048 identified as comprising 10 families (Laelapidae, Dermanyssidae, Macronyssidae, Aceocejidae, Ameroseiidae, Parasitidae, Parholaspidae, Macrochelidae, Pachylaelaptidae and Blattisocidae), 21 genera and 82 species (not including 847 unidentified individuals of gamasid mites due to structural damage, dirt cover (blurry structures) and some suspected new species). Of the 10 families, the family Laelapidae had the most abundant genera, species and individuals: 11 genera, 64 species and 24,853 individuals, which accounted for 95.41% of total gamasid mite individuals. The family Parholaspidae had the fewest genera, species and individuals (one species in one genus with only one individual), which accounted for less than 0.01% of total gamasid mite individuals (Table 2). The 82 identified gamasid mite species and their corresponding small mammal hosts in nine survey sites were given in “Appendix B: Taxonomic checklist of gamasid mites identified from nine survey sites in Three Parallel Rivers Area of northwest Yunnan, China (2001–2015)”.

### 3.2. Structural Characteristics and Species Diversity Changes of Gamasid Mite Communities

In this study, all the captured small mammal hosts and identified gamasid mites from the nine survey sites in the Three Parallel Rivers Area were taken as a host community unit and a gamasid mite community unit, respectively, then the species richness (*S*), Shannon–Wiener diversity index (*H*), Pielou evenness index (*E*) and Simpson dominance index (*D*) of the community structure were calculated. The identified 44 species and 3830 small mammal hosts constituted the “small mammal host community in the Three Parallel Rivers Area”. The species richness (*S*), Shannon–Wiener diversity (*H*), Pielou evenness (*E*) and Simpson dominance (*D*) of the host community were *S* = 44, *H* = 2.52, *E* = 0.67 and *D* = 0.12, respectively. As shown in Table 3, three species of hosts, *Apodemus chevrieri* (Milne-Edwards, 1868)*, Eothenomys miletus* (Thomas, 1914) and *A. draco* (Barrett-Hamiliton, 1900), were the most dominant in the Three Parallel Rivers Area, and their constituent ratios (*Cr*) were *Cr* = 24.28% (930/3830), *Cr* = 14.41% (552/3830) and *Cr* = 13.34% (511/3830), respectively. The individuals of *A. chevrieri*, *E. miletus* and *A. draco* exceeded 500, and the total constituent ratios of the three dominant hosts were 52.04% (1993/3830) of the whole. From *A. chevrieri*, 30 species and 1775 individuals of gamasid mites were collected, with 34 species and 877 individuals of gamasid mites from *E. miletus*, and 24 species and 558 individuals of gamasid mites from *A. draco*. The species richness was highest in *E. miletus* (*S* = 34), and Shannon–Wiener diversity was highest in *A. chevrieri* (*H* = 2.50) (Table 3). The species richness of the gamasid mite community on different host species is different, and the species composition of the dominant mites on different hosts is also quite different (Table 3 and Table 4).

The identified 82 species and 26,048 individuals (excluding unidentified ones) of gamasid mites constituted the “Gamasid mite community in the Three Parallel Rivers Area”. The species richness, Shannon–Wiener diversity, Pielou evenness and Simpson dominance of the gamasid mite community were *S* = 82, *H* = 2.33, *E* = 0.53 and *D* = 0.17, respectively. Three species of gamasid mites, *Dipolaelaps anourosorecis* (Gu et Wang 1981), *Laelaps nuttalli* Hirst, 1915 and *Laelaps echidninus* Berlese, 1887, were the most dominant, and their constituent ratios were *C_r_* = 32.14% (8371/26,048), *C_r_* = 20.97%, (5463/26,048) and *C_r_* = 11.35% (2957/26,048), respectively. The individuals of *D. anourosorecis*, *L. nuttalli* and *L. echidninus* were more than 2000, and the total constituent ratios of the three dominant gamasid mites were 64.46% (16,791/26,048) of the whole.

**Figure 2:**
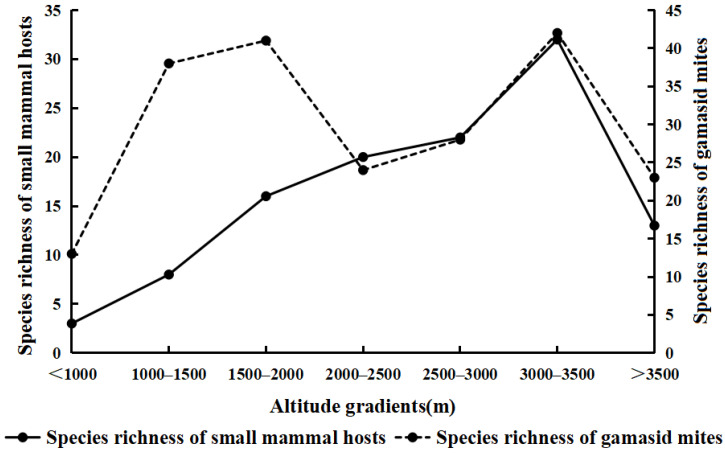
Species richness (number of species) variations of gamasid mites and their small mammal hosts along altitude gradients in Three Parallel Rivers Area in northwest Yunnan of China (2001–2015).

**Figure 3:**
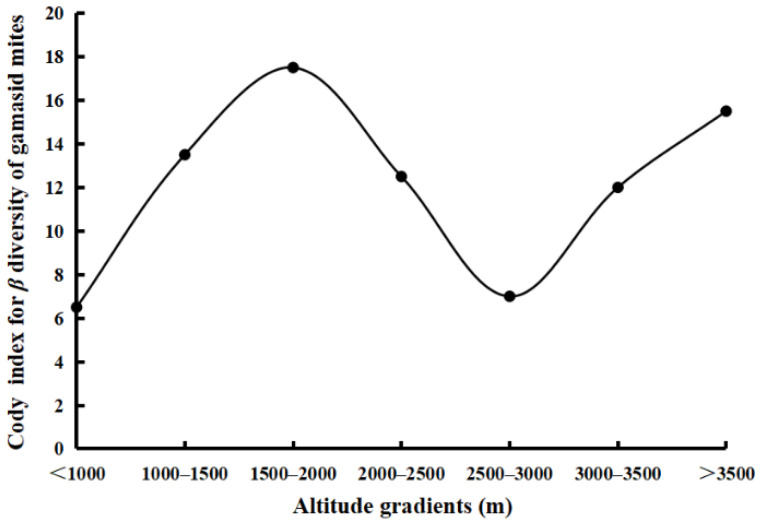
Variations of *β* diversity of gamasid mites along altitude gradients in Three Parallel Rivers Area in northwest Yunnan of China (2001–2015).

The altitudes of the nine survey sites in the Three Parallel Rivers Area are between 862 m and 3880 m. According to the recorded data, seven altitude gradients were used to analyze the variations of species diversity of gamasid mites and small mammal hosts along different altitude gradients. The species richness of small mammal hosts showed a tendency of increasing gradually in the beginning and then decreasing abruptly from the low altitude (<1000 m) to the high altitude (>3500 m) along the vertical gradients (Figure 2). And the species richness of small mammal hosts reached the highest value (*S* = 32) in the high-altitude regions between 3000 and 3500 m (Figure 2, Table 5). In addition, the species richness of gamasid mites formed the first peak (*S* = 41) in the low-altitude regions between 1500 and 2000 m and the second peak (*S* = 42) in the high-altitude regions between 3000 and 3500 m (Figure 2, Table 5). This showed that the changing trend of species richness of gamasid mites is not completely consistent with the small mammal hosts. Cody diversity index was used to analyze the beta-diversity (*β* diversity) of the gamasid mite community in the Three Parallel Rivers Area, and the *β* diversity of gamasid mites fluctuated from the low latitude to the high latitude of nine survey sites, which reached the highest value (*β* = 17.5) in the low-altitude regions between 1500 and 2000 m (Figure 3).

### 3.3. Species Abundance Distribution and Total Species Estimation of Gamasid Mite Community

**Figure 4:**
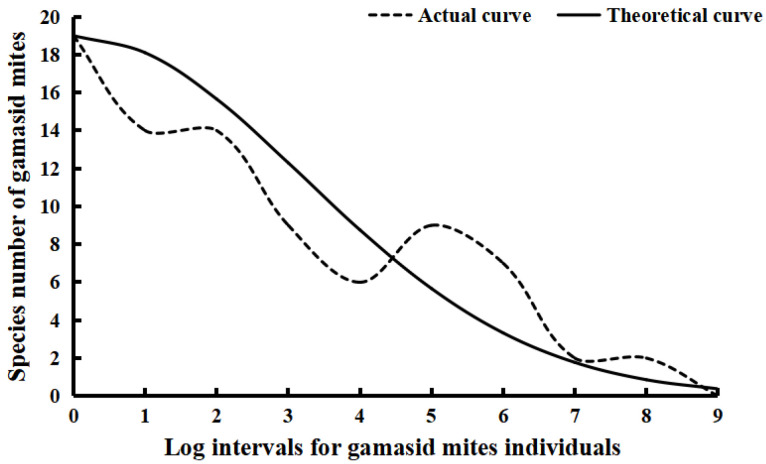
Theoretical curve fitting of species abundance distribution of gamasid mite community in Three Parallel Rivers Area in northwest Yunnan of China (2001–2015).

Of the 82 species and 26,048 individuals of identified gamasid mites, the number of gamasid mite individuals in the seventh to eighth logarithmic scale was the highest, but the number of species was minimal. When the logarithmic scale is zero (*R*_0_ = 0), the number of gamasid mites was only one individual, but the number of species was large (*S*_0_ = 19). Based on Preston’s lognormal model, the species abundance distribution of the gamasid mite community in the Three Parallel Rivers Area was successfully fitted by the lognormal distribution with *α* = 0.22 and *R*^2^ = 0.9879. The theoretical curve equation was $\hat{S}\left(R\right)=19e^{-{\left[0.22\left(R-0\right)\right]}^{2}}$(*S*_0_ = 19, *R*_0_ = 0). The species abundance distribution curve showed that the rare species of gamasid mites with few individuals accounted for the majority of species in the community, while the dominant species with a large number of individuals were only a few. With the increase of the number of gamasid mite individuals, the number of gamasid mite species gradually decreased (Table 6, Figure 4). Of the 82 gamasid mite species, 19 species had only one individual collected, 12 species had two individuals, and one species had three individuals. On the basis of the curve fitting, the total gamasid mite species in the Three Parallel Rivers Area was estimated to be 153 species (*S_T_* = 153), 71 species more than the actual collected 82 species.

## 4. Discussion

The present study showed a great species diversity of gamasid mites and their small mammal hosts in the Three Parallel Rivers Area of China. From abundant species of small mammal hosts (44 species with 3830 individuals), a total of 26,895 individuals of gamasid mites were collected, and 26,048 of them (26,048/26,895) are identified as comprising 82 species and 21 genera in ten families. The Three Parallel Rivers Area is only a partial area in the northwestern Yunnan Province, southwest China, in which the identified species of gamasid mites (82 species) in the present study were not only much more than those from other provinces of China, but also vastly exceeded those recorded from some other regions in the world. The identified 82 species of gamasid mites on small mammals in the present study were obviously greater than the total recorded species of the mites (excluding soil-living and free-living species) in other provinces of China. For example, there have been 53 gamasid mites species recorded from Zhejiang Province, 46 species from Hebei Province, 78 species from Hubei Province, 21 species from Chongqing municipality (a provincial administrative region), 28 species from Shandong Province and 69 species from Fujian province, respectively, according to some previous literature [48,49,50,51,52,53]. In the world, 56 species of gamasid mites were recorded from two geomorphologic complexes of South-East Slovakia: Košická kotlina basin and Východoslovenská rovina plain, 71 gamasid mite species in Asiatic Russia and 37 species in the Kurzeme Coast of Latvia (Eastern Europe) [54,55,56]. The present study involved only the ectoparasitic gamasid mites on small mammals (including facultatively and occasionally ectoparasitic species). If soil-living and free-living gamasid mites are investigated in the future, the number of species would be greatly increased. The abundance of gamasid mite species in the Three Parallel Rivers Area is related to the unique geographical environment of this area. Located in the longitudinal valley of the Hengduan Mountains, the complex and diverse variations in topographies, landscapes, ecological habitats and climate types, with huge elevation differences in the Three Parallel Rivers Area support the rich biological resources and high biodiversity together [20,57,58]. The lowest altitude of the Three Parallel Rivers Area is 720 m in Nujiang river valley, and the highest altitude is 6740 m on the top of the Meli Snow Mountains [22,59]. There are many high mountains with an altitude of more than 4000 m in the Three Parallel Rivers Area, such as Laojun Mountain, Gaoligong Mountain, Yulong Snow Mountain, Haba Snow Mountain and Meili Snow Mountain [25]. These mountain landforms usually have different vertical forest zones and climatic types, with high landscape heterogeneity and biodiversity, and the species composition of plants and animals are quite different from the bottom to the top of the mountains [20,57,58]. The species diversity of ectoparasitic gamasid mites is closely related to that of their small mammal hosts. The above complex geographical, topographical and ecological environments led to the abundant species of small mammal hosts (44 species) and gamasid mites (82 species) in the present study. The unique geographical, topographical and ecological characteristics should be the initial factors influencing the high species diversity of gamasid mite species in the Three Parallel Rivers Area.

Due to the limitations of transportation, human resources and financial funds, the nine survey sites (elevations between 862 m and 3880 m) in this study have not covered all the mountain landforms in the Three Parallel Rivers Area. The alpine zone above 4000 m has not been involved, and the number of survey sites is also limited (only nine sites). This study is only a preliminary report on gamasid species diversity and community characteristics in the Three Parallel Rivers Area of China. With the continuous expansion and deepening of field investigation in the future, more small mammal hosts and gamasid mite species will be collected, and the composition of dominant hosts and gamasid mite species may also change to some extent. The results of this study suggest that there are abundant species of small mammal hosts and gamasid mites with a complex community structure and high species diversity in the Three Parallel Rivers Area of China. Moreover, the results show that further research is of great academic value.

The variation of species diversity along elevation gradients has been one crucial topic in ecology and biodiversity research in recent years [60,61,62]. The variation patterns of species diversity along elevation gradients are often diverse in different geographical regions because of habitat heterogeneity, vegetation difference, climate instability and some other environmental factors [62,63,64]. Some previous studies showed that the species diversity of lizards decreases with increasing elevation and it peaks at middle elevations [65]. The highest species diversity of chigger mites also occurred in the intermediate altitude regions (2000–2500 m and 2000–3000 m) [41,66,67]. In this study, species richness and *β* diversity are used to analyze the variation of gamasid mite species diversity along altitudinal gradients. The results of this study showed that the species diversity of the gamasid mite community in the Three Parallel Rivers Area fluctuated significantly in different elevation gradients. The peak of species richness (*S* = 42) occurred at the altitude between 3000 and 3500 m, and the peak of *β* diversity (*β* = 17.5) occurred at the altitude between 1500 and 2000 m (Figure 2 and Figure 3). The result in this paper is not completely consistent with the previous studies [41] and we need further studies.

In this paper, the species composition, species diversity and dominant species of gamasid mites on different host species were also significantly different (Table 3 and Table 4), which reflects the heterogeneity of gamasid mite communities on different hosts. The species diversity variation and community heterogeneity of gamasid mites on different host species may be associated with different biological characteristics of different host species and the low host specificity of gamasid mites. Different species of animal hosts with different biological characteristics usually have different susceptibility to parasite infections (including ectoparasite infestations) with different parasite burdens, species composition and species diversity [40,67,68,69]. Many ectoparasitic gamasid mites have low host specificity, and cross infestation of the mites among different host species is very common, which may also lead to the heterogeneity of a gamasid mite community on small mammals [67,70,71]. The species diversity variation and community heterogeneity of gamasid mites on different host species further suggests the extraordinary complexity of gamasid mite communities on small mammals in the Three Parallel Rivers Area of China.

Of the 82 identified gamasid mite species in this study, several species are closely related to medicine. *Ornithonyssus bacoti* (Hirst, 1913) often stings humans to cause dermatitis [5,7,72], and it is an effective vector of rickettsialpox and an intermediate host of cotton rat filaria, *L. carinii* [17,18,72]. Moreover, *O. bacoti* has been proved to be an important potential vector of hantavirus, the causative agent of hemorrhagic fever with renal syndrome, HFRS [2,11,12,13]. Aside from *O. bacoti*, some other species of gamasid mites found in the present study, e.g., *Haemolaelaps glasgowi* (Ewing, 1925), *Tricholaelaps myonysognathus* (Grochovskaya et Nguen-Xuan-Xoe, 1961), *Eulaelaps stabularis* Koch, 1836, *Eulaelaps shanghaiensis* Wen, 1976 and *L. echidninus* can also invade humans to cause dermatitis, and they can be the potential vectors of hantavirus as well [3,14,73]. The occurrence of the above mite species in the Three Parallel Rivers Area may increase the potential danger to the people’s health in this area.

Species abundance distribution, together with the fitting of its theoretical curve, is an important issue in community ecology, and it describes the relationship between the species and individuals in a specific community, that is, the abundance distribution of different species in the community. The species abundance distribution can directly reflect the proportion structure of the dominant, common and rare species in a community [44,74]. Based on the theoretical curve fitting of species abundance distribution, the expected total number of species (the theoretical total number of species) can be roughly estimated [45,75,76]. The species abundance distribution of gamasid mites in the present study was successfully fitted by Preston’s lognormal distribution model, suggesting that it conforms to the lognormal distribution. The result showed that the expected number of gamasid mite species showed a gradual descending tendency with the increase of gamasid mite individuals. It reveals that the majority of the gamasid mite species are sporadic with few individuals, and few gamasid mite species are dominant with abundant individuals. The tendency of species abundance distribution in this paper is almost consistent with that in other ectoparasite communities, such as chigger mites in the previous papers [71,77].

How to scientifically estimate the expected total number of species (the theoretical total number of species) in a specific community is also an important issue in ecology and several methods have been previously used to estimate the total number of species [40,77,78]. As one estimation method, the method used in the present study is based on the curve fitting of species abundance, and it has been applied to study the community of chigger mites and some other ectoparasites, such as fleas and sucking lice [44,47]. The result showed that the theoretical total number of gamasid mite species in the Three Parallel Rivers Area is 153 (*S_T_* = 153), which greatly exceeded the 82 actually identified species (*S_A_* = 82). It reveals the fact that there are still many gamasid mite species (*S_M_* = 71) that have not been collected and which were missed in the actual field investigation. The results further suggest that gamasid mites on small mammals in the Three Parallel Rivers Area of China are abundant with complex community structures and high species diversity, which is of great academic value for further in-depth study.

## 5. Conclusions

Gamasid mites on small mammals are abundant with complex community structures and high species diversity in the Three Parallel Rivers Area of China. There is an apparent community heterogeneity of the mites on different hosts and in different environments.

## Figures and Tables

**Table 1:** Identified small mammal hosts from nine survey sites in Three Parallel Rivers Area in northwest Yunnan of China (2001–2015).

Order Names of Small Mammal Hosts	Individuals of Hosts	Number of Host Families	Number of Host Genera	Number of Host Species	Constituent Ratios *C_r_* (%)
Rodentia	3517	4	18	33	91.83
Eulipotyphla	290	2	6	7	7.57
Scandentia	11	1	1	1	0.29
Lagomorpha	11	1	1	2	0.29
Carnivora	1	1	1	1	0.03
Total	3830	9	27	44	100.00

**Table 2:** Identified gamasid mites from nine survey sites in Three Parallel Rivers Area in northwest Yunnan of China (2001–2015).

Family Names of Gamasid Mites	Number of Gamasid Mite Genera	Number of Gamasid Mite Species	Individuals of Gamasid Mites	Constituent Ratios of Gamasid Mite Individuals *C_r_* (%)
Laelapidae	11	64	24,853	95.41
Dermanyssidae	1	1	16	0.06
Macronyssidae	1	1	928	3.56
Aceosejidae	1	6	31	0.12
Ameroseiidae	2	2	4	0.02
Parasitidae	1	2	3	0.01
Parholaspidae	1	1	1	0.01
Macrochelidae	1	2	48	0.18
Pachylaelaptidae	1	2	11	0.04
Blattisocidae	1	1	153	0.58
Total	21	82	26,048	100.00

**Table 3:** Statistics on community structure of gamasid mites on three dominant species of small mammals in Three Parallel Rivers Area in northwest Yunnan of China (2001–2015).

Names of Dominant Hosts	Number of Hosts	*C_r_* of Hosts	Individuals of Gamasid Mites	*S*	*H*	*E*	*D*
*Apodemus chevrieri* (Milne-Edwards, 1868)	930	24.28%	1775	30	2.50	0.73	0.12
*Eothenomys miletus* (Thomas, 1914)	552	14.41%	877	34	1.79	0.51	0.36
*Apodemus draco* (Barrett-Hamiliton, 1900)	511	13.34%	558	24	2.24	0.70	0.18

**Table 4:** Dominant species of gamasid mites on three dominant species of small mammals in Three Parallel Rivers Area in northwest Yunnan of China (2001–2015).

Names of Dominant Host Species	Names of Dominant Gamasid Mite Species	Individuals of Total Gamasid Mites	Individuals of Dominant Gamasid Mite Species	*C_r_* of Dominant Gamasid Mite Species
*Apodemus chevrieri*	*Eulaelaps shanghaiensis* Wen, 1976	1775	493	27.77%
*Eothenomys miletus*	*Laelaps chini* Wang et Li, 1965	877	509	58.04%
*Apodemus draco*	*Laelaps jingdongensis* Tian, Duan et Fang, 1990	558	207	37.10%

**Table 5:** Statistics on community structure of small mammal hosts and gamasid mites along different altitude gradients in Three Parallel Rivers Area in northwest Yunnan of China (2001–2015).

Altitude Gradients (m)	Individuals of Small Mammal Hosts	Individuals of Gamasid Mites	Statistics on Community Structure of Small Mammal Hosts				Statistics on Community Structure of Gamasid Mites			
			*S*	*H*	*E*	*D*	*S*	*H*	*E*	*D*
<1000	182	3702	3	0.39	0.36	0.81	13	0.85	0.33	0.55
1000–1500	214	7315	8	1.47	0.71	0.30	38	1.84	0.51	0.22
1500–2000	400	8710	16	1.91	0.69	0.20	41	1.16	0.31	0.52
2000–2500	367	1498	20	2.18	0.73	0.18	24	2.02	0.64	0.21
2500–3000	735	1304	22	1.67	0.54	0.33	28	2.59	0.78	0.10
3000–3500	1799	2954	32	2.17	0.63	0.16	42	2.61	0.70	0.10
>3500	133	565	13	1.69	0.66	0.26	23	2.07	0.66	0.19

**Table 6:** The fitting results of species abundance distribution of gamasid mite community in Three Parallel Rivers Area in northwest Yunnan of China (2001–2015).

Log Intervals Based on log_3_M	Individual Ranges of Gamasid Mites in Each Log Interval	Midpoint Values of Each Individual Range	Actual Gamasid Mite Species	Theoretical Gamasid Mite Species
0	0–1	1	19	19.00
1	2–4	3	14	18.10
2	5–13	9	14	15.66
3	14–40	27	9	12.29
4	41–121	81	6	8.76
5	122–364	243	9	5.67
6	365–1093	729	7	3.33
7	1094–3280	2187	2	1.77
8	3281–9841	6561	2	0.86

## Data Availability

The experimental data used to support the findings of this study are available from the corresponding author request.

## Appendix A. Taxonomic Checklist of Small Mammals Captured from Nine Survey Sites in Three Parallel Rivers Area of Northwest Yunnan, China (2001–2015)

**Orders, Families, Genera and Species of Small Mammal Hosts**	**Survey Sites**	**Individuals of Hosts**
Order Rodentia		
Ⅰ Family Muridae		
1 *Rattus tanezumi* (Temminck, 1844)	Gongshan, Jianchuan, Yulong, Weixi, Lushui, Fugong	244
2 *R. nitidus* (Hodgson, 1845)	Gongshan, Jianchuan, Yulong, Weixi, Deqin, Lushui, Fugong	129
3 *R. norvegicus* (Berkenhout, 1769)	Gongshan, Jianchuan, Yulong, Weixi	136
4 *R. andamanensis* (Blyth, 1860)	Gongshan, Jianchuan, Deqin, Lushui	37
5 *Niviventer confucianus* (Milne-Edwards, 1871)	Gongshan, Jianchuan, Xianggelila, Weixi, Deqin	232
6 *N. fulvescens* (Gray, 1847)	Gongshan, Weixi, Fugong	101
7 *N. andersoni* (Thomas, 1911)	Gongshan, Jianchuan, Lanping, Weixi	35
8 *N. excelsior* (Thomas 1911)	Deqin	2
9 *Mus musculus* Linnaeus, 1758	Gongshan, Jianchuan	9
10 *M. caroli* Bonhote, 1902	Yulong	1
11 *Apodemus draco* (Barrett-Hamiliton, 1900)	Gongshan, Weixi	511
12 *A. peninsulae* (Thomas, 1907)	Gongshan, Weixi	91
13 *A. chevrieri* (Milne-Edwards, 1868)	Gongshan, Jianchuan, Yulong, Lanping, Weixi, Xianggelila	930
14 *A. agrarius* (Pallas, 1771)	Yulong, Xianggelila	4
15 *A. latronum* Thomas, 1911	Gongshan, Yulong, Xianggelila, Lanping, Deqin	335
16 *Berylmys bowersi* (Anderson, 1879)	Gongshan, Weixi	9
17 *Micromys minutus* (Pallas, 1771)	Jianchuan	9
18 *Vernaya fulva* (G.M. Allen, 1927)	Weixi	1
19 *Bandicota indica* (Bechstein, 1800)	Weixi	1
II Family Cricetidae		
20 *Eothenomys miletus* (Thomas, 1914)	Gongshan, Jianchuan, Yulong, Lanping, Weixi, Deqin	552
21 *E. eleusis* (Thomas, 1911)	Gongshan, Lanping	13
22 *E. proditor* Hinton, 1923	Yulong, Xianggelila	23
23 *E. custos* (Thomas, 1912)	Yulong, Lanping	30
Ⅲ Family Sciuridae		
24 *Callosciurus quinquestriatus* Anderson, 1871	Lanping, Weixi	6
25 *Tamiops swinhoei* (Milne-Edwards, 1874)	Gongshan	3
26 *Dremomys pernyi* (Milne-Edwards, 1867)	Jianchuan, Weixi	20
27 *Rupestes forresti* (Thomas, 1922)	Jianchuan	13
Ⅳ Family Pteromyidae		
28 *Petaurista albiventer* (Gray, 1834)	Gongshan, Weixi	4
29 *P. xanthotis* (Milne-Edwards, 1872)	Weixi	2
30 *Trogopterus xanthipes* (Milne-Edwards, 1867)	Gongshan, Weixi	18
31 *Belomys pearsonii* (Gray, 1842)	Weixi	1
32 *Hylopetes alboniger* (Hodgson, 1836)	Weixi	4
33 *Pteromys volans* (Linnaeus, 1758)	Weixi	11
Order Eulipotyphla		
Ⅴ Family Talpidae		
34 *Scaptonyx fusicaudus* (Milne-Edwrds, 1872)	Weixi	1
VI Family Soricidae		
35 *Sorex excelsus* (G.M. Allen, 1923)	Gongshan	6
36 *S. cylindricauda* (Milne-Edwrds, 1872)	Weixi	3
37 *Episoriculus leucops* (Hosfield, 1855)	Gongshan, Weixi	7
38 *Suncus murinus* (Linnaeus, 1766)	Yulong, Xianggelila, Weixi	14
39 *Crocidura attenuata* (Milne-Edwards, 1872)	Jianchuan, Yulong	32
40 *Anourosorex squamipes* (Milne-Edwards, 1872)	Gongshan, Lanping, Weixi	227
Order Scandentia		
VII Family Tupaiidae		
41 *Tupaia belangeri* (Wagner, 1841)	Yulong	11
Order Lagomorpha		
VIII Family Ochotonidae		
42 *Ochotona thibetana* (Milne-Edwards, 1871)	Gongshan, Lanping, Weixi	10
43 *O. roylii* (Ogilby, 1839)	Gongshan	1
Order Carnivora		
IX Family Mustelidea		
44 *Mustela kathiah* Hodgson, 1835	Yulong	1

## Appendix B. Taxonomic Checklist of Gamasid Mites Identified from Nine Survey Sites in Three Parallel Rivers Area of Northwest Yunnan, China (2001–2015)

**Families, Genera and Species of Gamasid Mites**	**Corresponding Small Mammal Hosts**	**Survey Sites**
I Family Laelapidae Berlese 1892		
1 *Laelaps nuttalli* Hirst, 1915	*Rattus tanezumi*, *Eothenomys miletus*, *R. nitidus*, *R. andamanensis*, *Apodemus chevrieri*, *R. norvegicus*, *A. draco*, *Niviventer confucianus*, *A. latronum*, *N. andersoni*, *Ochotona thibetana*, *E. custos*, *N. fulvescens*, *Mus musculus*, *Anourosorex squamipes*, *A. peninsulae*, *A. agrarius*	Gongshan, Lushui, Fugong, Jianchuan, Yulong, Weixi, Xianggelila
2 *L. echidninus* Berlese, 1887	*R. tanezumi*, *E. miletus*, *R. nitidus*, *R. andamanensis*, *N. confucianus*, *A. squamipes*, *R. norvegicus*, *N. fulvescens*, *Berylmys bowersi*, *A. chevrieri*, *Micromys minutus*	Deqin, Lushui, Fugong, Gongshan, Jianchuan, Yulong, Weixi
3 *L. guizhouensis* Gu *et* Wang, 1981	*A. chevrieri*, *M. musculus*	Gongshan, Jianchuan, Yulong
4 *L. turkestanicus* Lange, 1955	*N. confucianus*, *N. fulvescens*, *N. andersoni*, *A. chevrieri*, *N. excelsior*, *R. tanezumi*, *R. andamanensis*, *E. miletus*, *R. norvegicus*, *M. musculus*, *R. nitidus*, *Dremomys pernyi*, *M. caroli*, *B. bowersi*, *Petaurista albiventer*, *A. draco*, *A. peninsulae*, *Pteromys volans*, *P. xanthotis*, *Vernaya fulva*, *Trogopterus xanthipes*	Deqin, Fugong, Gongshan, Jianchuan, Yulong, Lanping, Weixi, Xianggelila
5 *L. traubi* Domrow, 1962	*N. andersoni*, *E. miletus*, *N. excelsior*, *R. nitidus*, *N. fulvescens*, *N. confucianus*, *A. chevrieri*, *A. latronum*, *A. draco*, *R. norvegicus*, *D. pernyi*, *P. volans*, *P. xanthotis*, *B. bowersi*, *Episoriculus leucops*, *Bandicota indica*,	Deqin, Fugong, Gongshan, Jianchuan, Yulong, Lanping, Weixi, Xianggelila
6 *L. chini* Wang *et* Li, 1965	*E. miletus*, *A. squamipes*, *N. confucianus*, *E. custos*, *E. eleusis*, *A. chevrieri*, *R. tanezumi*, *Suncus murinus*, *A. latronum*, *A. draco*, *E. leucops*, *M. minutus*, *R. norvegicus*, *E. proditor*, *A. peninsulae*	Gongshan, Deqin, Jianchuan, Yulong, Lanping, Weixi, Xianggelila
7 *L. paucisetosa* Gu *et* Wang, 1981	*N. fulvescens*	Gongshan
8 *L. algericus* Hirst, 1925	*M. caroli*,	Yulong
9 *L. xingyiensis* Gu *et* Wang, 1981	*M. minutus*,	Jianchuan
10 *L. fukienensis* Wang, 1963	*N. confucianus*, *N. fulvescens*, *N. andersoni*, *N. excelsior*, *M. musculus*, *R. nitidus*,	Deqin, Fugong, Gongshan, Lanping
11 *L. liui* Wang *et* Li, 1965	*B. bowersi*	Gongshan
12 *L. jettmari* Vitzthum, 1930	*A. chevrieri*, *E. miletus*, *M. minutus*, *R. norvegicus*, *A. latronum*, *E. eleusis*,	Gongshan, Jianchuan, Yulong, Lanping, Xianggelila
13 *L. hongaiensis* Grochovskaya *et* Nguen-Xuan-Xoe, 1961	*N. confucianus*,	Gongshan
14 *L. jingdongensis* Tian, Duan *et* Fang, 1990	*A. draco*, *E. miletus*, *A. chevrieri*, *A. latronum*, *E. eleusis*, *Callosciurus quinquestriatus*, *N. confucianus*, *A. peninsulae*, *R. norvegicus*, *D. pernyi*, *E. leucops*	Gongshan, Deqin, Lanping, Weixi
15 *Haemolaelaps glasgowi* (Ewing, 1925)	*A. chevrieri*, *A. squamipes*, *N. fulvescens*, *P. albiventer*, *R. norvegicus*, *E. miletus*, *M. musculus*, *E. proditor*	Gongshan, Jianchuan, Weixi, Xianggelila
16 *H. casalis* (Berlese, 1887)	*A. chevrieri*, *N. confucianus*, *E. miletus*, *T. xanthipes*, *P. volans*,	Gongshan, Weixi
17 *H. petauristae* Gu *et* Wang, 1980	*T. xanthipes*	Gongshan
18 *H. anomalis* Wang, Liao *et* Lin, 1981	*B. bowersi*	Gongshan
19 *Androlaelaps singularis* Wang *et* Li, 1965	*A. chevrieri*, *A. squamipes*, *R. nitidus*, *R. norvegicus*, *R. andamanensis*, *S. murinus*,	Fugong, Gongshan, Jianchuan, Yulong, Xianggelila
20 *Dipolaelaps anourosorecis* (Gu *et* Wang 1981)	*E. miletus*, *R. norvegicus*, *Crocidura attenuata*, *A. squamipes*, *A. draco*, *Sorex excelsus*, *N. andersoni*, *R. nitidus*, *E. leucops*, *O. roylii*, *A. chevrieri*, *R. andamanensis*, *B. bowersi*, *P. albiventer*, *E. custos*,	Gongshan, Yulong
21 *D. jiangkouensis* Gu, 1985	*N. confucianus*, *N. fulvescens*,	Gongshan
22 *D. longisetosus* Huang, 1985	*A. latronum*	Deqin
23 *Tricholaelaps myonysognathus* (Grochovskaya *et* Nguen-Xuan-Xoe, 1961)	*R. tanezumi*,	Lushui
24 *Hypoaspis pavlovskii* (Bregetova, 1956)	*N. andersoni*, *E. miletus*, *A. draco*, *N. confucianus*, *A. squamipes*, *A. chevrieri*, *R. tanezumi*, *N. fulvescens*, *R. nitidus*, *R. andamanensis*, *S. murinus*, *R. norvegicus*, *A. latronum*, *A. peninsulae*	Gongshan, Lushui, Fugong, Jianchuan, Yulong, Lanping, Weixi, Xianggelila
25 *H. miles* (Berlese, 1892)	*R. tanezumi*, *A. squamipes*, *E. miletus*, *R. nitidus*, *A. chevrieri*,	Gongshan, Jianchuan, Yulong, Xianggelila
26 *H. lubrica* Voigts *et* Oudemans, 1904	*R. norvegicus*, *R. nitidus*, *A. latronum*, *R. tanezumi*, *N. fulvescens*, *A. chevrieri*, *C. attenuata*,	Gongshan, Deqin, Lushui, Fugong, Yulong
27 *H. chianensis* Gu, 1990	*R. andamanensis*, *A. chevrieri*	Gongshan
28 *H. praesternalis* Willmann, 1949	*A. squamipes*, *R. tanezumi*, *R. nitidus*,	Gongshan, Lushui, Fugong
29 *H. concinna* (Teng, 1982)	*R. nitidus*, *R. tanezumi*, *R. andamanensis*, *N. fulvescens*,	Lushui, Fugong, Gongshan
30 *H. aculeifer* (Canestrini, 1884)	*R. nitidus*	Fugong
31 *H. ovatus* Ma, Ning *et* Wei, 2003	*R. norvegicus*, *A. draco*, *A. squamipes*, *R. nitidus*	Gongshan
32 *H. digitalis* Teng, 1981	*A. squamipes*	Gongshan
33 *H. tengi* Gu *et* Bai, 1991	*A. squamipes*,	Gongshan
34 *Haemogamasus oliviformis* Teng *et* Pan, 1964	*A. draco*, *A. squamipes*, *E. miletus*, *E. eleusis*, *O. thibetana*, *A. chevrieri*, *R. nitidus*, *R. andamanensis*, *N. fulvescens*, *A. latronum*, *N. confucianus*, *A. peninsulae*, *R. norvegicus*, *D. pernyi*, *C. quinquestriatus*, *E. custos*, *A. agrarius*	Gongshan, Deqin, Jianchuan, Yulong, Lanping, Weixi, Xianggelila
35 *H. dorsalis* Teng *et* Pan, 1964	*E. custos*, *A. chevrieri*	Yulong, Lanping
36 *H. gongshanensis* Tian *et* Gu, 1989	*E. miletus*, *A. squamipes*, *A. latronum, A. draco*, *N. confucianus*, *O. thibetana*	Gongshan, Deqin, Weixi
37 *H. monticola* Wang *et* Li, 1965	*A. draco*, *A. squamipes*, *R. andamanensis*, *N. fulvescens*, *R. nitidus*, *N. confucianus*, *S. murinus, A. chevrieri*, *E. custos*, *A. latronum*, *S. excelsus*	Fugong, Gongshan, Yulong, Lanping, Weixi, Xianggelila
38 *H. sexsetosus* Guo *et* Gu, 1993	*N. fulvescens*, *E. miletus*, *T. xanthipes*	Gongshan, Jianchuan
39 *H. pontiger* (Berlese, 1903)	*R. norvegicus*	Weixi
40 *H. multidentis* Guo *et* Gu, 1997	*E. miletus*	Weixi
41 *H. quadrisetatus* Vitzthum, 1926	*A. draco*, *A. squamipes*, *N. confucianus*, *R. norvegicus*	Gongshan, Weixi
42 *H. yunlongensis* Gu *et* Fang, 1987	*E. miletus*	Weixi
43 *H. paradauricus* Teng *et* Pan, 1964	*A. chevrieri*, *N. confucianus*	Lanping, Weixi
44 *H. sanxiaensis* Liu *et* Ma, 2001	*A. squamipes*, *P. volans*, *T. xanthipes*	Weixi
45 *H. hodosi* Buiakova *et* Goncharova, 1961	*E. miletus*, *Scaptonyx fusicaudus*	Weixi
46 *H. trifurcisetus* Zhou *et* Jiang, 1987	*R. nitidus*,	Gongshan
47 *Eulaelaps stabularis* Koch, 1836	*R. nitidus*, *N. fulvescens*, *R. tanezumi*, *E. proditor*	Fugong, Lushui, Xianggelila
48 *E. shanghaiensis* Wen, 1976	*A. draco*, *E. miletus*, *N. confucianus, A. chevrieri*, *A. latronum*	Weixi, Gongshan, Jianchuan, Yulong, Xianggelila
49 *E. dremomydis* Gu *et* Wang, 1984	*Rupestes forresti*, *D. pernyi*, *E. miletus*, *A. latronum*, *N. confucianus*, *A. peninsulae*	Jianchuan, Lanping, Weixi
50 *E. substabularis* Yang *et* Gu, 1986	*A. draco*, *A. squamipes*, *R. tanezumi*, *A. latronum*, *A. peninsulae*, *E. miletus*, *R. norvegicus*, *R. nitidus*, *N. fulvescens*, *A. chevrieri*, *A. agrarius*, *E. eleusis*, *O. thibetana*, *N. confucianus*, *T. xanthipes*, *Hylopetes alboniger*	Gongshan, Deqin, Fugong, Jianchuan, Yulong, Lanping, Weixi
51 *E. huzhuensis* Yang *et* Gu, 1985	*A. latronum*, *R. norvegicus*, *R. nitidus*, *A. chevrieri*, *A. draco*, *E. miletus*, *N. confucianus*, *A. peninsulae*, *S. cylindricauda*, *E. leucops*, *P. volans*, *T. xanthipes*, *Belomys pearsonii*	Deqin, Fugong, Gongshan, Lushui, Weixi
52 *E. silvestris* Zhou, 1981	*R. tanezumi*,	Fugong
53 *E. dongfangis* Wen, 1976	*R. nitidus*	Fugong
54 *Gymnolaelaps sinensis* Wang, Zhou *et* Ji, 1991	*A. chevrieri*	Weixi
55 *G. weishanensis* Gu *et* Guo, 1997	*N. fulvescens*, *A. chevrieri*,	Gongshan, Jianchuan, Yulong
56 *Cosmolaelaps retirugi* (Ma, Yang *et* Zhang, 2004)	*A. squamipes*, *R. norvegicus*, *E. miletus*	Gongshan, Weixi
57 *C. yeruiyuae* Ma, 1995	*A. squamipes*, *R. tanezumi*, *R. nitidus*, *N. fulvescens*,	Gongshan, Lushui, Fugong
58 *Hirstionyssus sunci* Wang, 1962	*E. miletus*, *A. squamipes*, *A. chevrieri*, *R. norvegicus*, *R. tanezumi*, *R. nitidus*, *N. confucianus*, *A. latronum*, *C. attenuata*, *Mustela kathiah*, *Tupaia belangeri*, *A. draco*, *A. agrarius*, *R. andamanensis*,	Gongshan, Deqin, Fugong, Jianchuan, Yulong, Lanping, Weixi, Xianggelila
59 *H. callosciuri* Bregetova *et* Grokhovskaya, 1961	*Tamiops swinhoei*	Gongshan
60 *H. neosinicus* Teng *et* Pan, 1962	*A. draco*, *E. miletus*, *N. confucianus*	Weixi
61 *H. qinghaiensis* Gu *et* Yang, 1986	*E. miletus*	Weixi
62 *H. microti* Hsu *et* Ma, 1964	*E. miletus*	Weixi
63 *H. isabellinus* (Oudemans, 1913)	*A. draco*, *E. miletus*, *A. chevrieri*	Weixi
64 *H. musculi* (Johnston, 1849)	*A. latronum*	Deqin
II Family Dermanyssidae Kolenati 1859		
65 *Liponyssoides muris* (Hirst, 1913)	*R. norvegicus*, *T. belangeri*	Jianchuan, Yulong
III Family Macronyssidae Oudemans 1996		
66 *Ornithonyssus bacoti* (Hirst, 1913)	*R. tanezumi*, *R. nitidus*, *R. norvegicus*, *R. andamanensis*, *A. draco*, *N. confucianus*, *A. chevrieri*, *A. peninsulae*, *N. andersoni*	Deqin, Lushui, Fugong, Gongshan, Weixi
IV Family Aceosejidae Baker *et* Wharton 1952		
67 *Lasioseius medius* Gu *et* Guo, 1994	*A. squamipes*, *E. miletus*, *E. custos*	Gongshan, Yulong
68 *L. trifurcipilus* Gu *et* Guo, 1996	*A. chevrieri*	Xianggelila
69 *L. qinghaiensis* Wang *et* Li, 2001	*A. squamipes*, *E. miletus*, *R. nitidus*	Gongshan
70 *L. multispathus* Gu *et* Huang, 1990	*A. squamipes*	Gongshan
71 *L. liaohaorongae* Ma, 1996	*A. squamipes*	Gongshan
72 *L. chenpengi* Ma *et* Yin, 1999	*A. squamipes*	Gongshan
V Family Ameroseiidae Evans 1963		
73 *Ameroseius taoerhensis* Ma, 1995	*A. latronum*	Deqin
74 *Sinoseius lobatus* Bai *et* Gu, 1995	*E. miletus*, *D. pernyi*	Weixi
VI Family Parasitidae Oudemans 1901		
75 *Parasitus consanguineus* Oudemans *et* Voigts, 1904	*A. draco*	Gongshan
76 *P. wangdunqingi* Ma, 1995	*A. squamipes*	Gongshan
VII Family Parholaspidae Evans 1956		
77 *Gamasholaspis eothenomydis* Gu, 1984	*A. squamipes*	Gongshan
VIII Family Macrochelidae Vitzthum, 1930		
78 *Macrocheles liguizhenae* Ma, 1996	*A. draco*, *A. squamipes*, *S. excelsus*, *E. miletus*, *R. nitidus*	Gongshan, Fugong
79 *M. muscaedomesticae* (Scopoli, 1772)	*A. draco*, *A. squamipes*, *E. miletus*, *R. norvegicus*	Gongshan
IX Family Pachylaelaptidae Berlese 1888		
80 *Pachylaelaps badongensis* (Liu *et* Ma, 2003)	*A. draco*	Weixi
81 *P. nuditectus* Ma *et* Yin, 2000	*A. latronum*	Deqin
X Family Blattisocidae Garman, 1948		
82 *Proctolaelaps pygmaeus* (Muller, 1859)	*R. tanezumi*, *N. confucianus*, *N. fulvescens*, *A. draco*, *E. miletus*, *A. peninsulae*, *R. norvegicus*, *P. xanthotis*, *R. nitidus*, *A. chevrieri*, *M. musculus*, *C. attenuata*, *A. agrarius*	Lushui, Fugong, Gongshan, Jianchuan, Yulong, Weixi, Xianggelila

## References

[1] Yin S.G., Bei N.X., Chen W.P. (2013). Soil Gamasida from Northeast China.

[2] Xiang R., Guo X.G., Zhao C.F., Fan R., Mao K.Y., Zhang Z.W., Huang X.B. (2021). Infestation and distribution of gamasid mites on Himalayan field rat (*Rattus nitidus*) in Yunnan Province of Southwest China. Biologia.

[3] Huang L.Q., Guo X.G., Speakman J.R., Dong W.G. (2013). Analysis of gamasid mites (Acari: Mesostigmata) associated with the Asian house rat, *Rattus tanezumi* (Rodentia: Muridae) in Yunnan Province, southwest China. Parasitol. Res..

[4] Deng G.F., Teng K.F. (1993). Economic Insect Fauna of China Fasc. 40 Acari: Dermanyssoidese.

[5] Rosen S., Yeruham I., Braverman Y. (2002). Dermatitis in humans associated with the mites *Pyemotes tritici*, *Dermanyssus gallinae*, *Ornithonyssus bacoti* and *Androlaelaps casalis* in Israel. Med. Vet. Entomol..

[6] George D.R., Finn R.D., Graham K.M., Mul M.F., Maurer V., Moro C.V., Sparagano O.A. (2015). Should the poultry red mite *Dermanyssus gallinae* be of wider concern for veterinary and medical science?. Parasite Vector.

[7] Beck W., Fölster-Holst R. (2009). Tropical rat mites (*Ornithonyssus bacoti*)—Serious ectoparasites. J. Dtsch. Dermatol. Ges..

[8] Lopatina Iu V., Petrova A.D., Timoshkov V.V. (1998). The gamasid mites (Parasitiformes: Mesostigmata) of small mammals from undeveloped land in Moscow. Parazitologiia.

[9] Lucky A.W., Sayers C., Argus J.D., Lucky A. (2001). Avian mite bites acquired from a new source--pet gerbils: Report of 2 cases and review of the literature. Arch. Dermatol..

[10] Goldman L. (1948). Lichen urticatus syndrome as a manifestation of sensitivity to bites from various species of arthropods. Arch. Derm. Syphilol..

[11] Yin P.W., Guo X.G., Jin D.C., Fan R., Zhao C.F., Zhang Z.W., Huang X.B., Mao K.Y. (2021). Distribution and Host Selection of Tropical Rat Mite, *Ornithonyssus bacoti*, in Yunnan Province of Southwest China. Animals.

[12] Yin P.W., Guo X.G., Jin D.C., Song W.Y., Zhang L., Zhao C.F., Fan R., Zhang Z.W., Mao K.Y. (2021). Infestation and Seasonal Fluctuation of Gamasid Mites (Parasitiformes: Gamasida) on Indochinese Forest Rat, *Rattus andamanensis* (Rodentia: Muridae) in Southern Yunnan of China. Biology.

[13] Reeves W.K., Loftis A.D., Szumlas D.E., Abbassy M.M., Helmy I.M., Hanafi H.A., Dasch G.A. (2007). Rickettsial pathogens in the tropical rat mite *Ornithonyssus bacoti* (Acari: Macronyssidae) from Egyptian rats (*Rattus* spp.). Exp. Appl. Acarol..

[14] Yu X.J., Tesh R.B. (2014). The role of mites in the transmission and maintenance of Hantaan virus (Hantavirus: Bunyaviridae). J. Infect. Dis..

[15] Jiang F.C., Wang L., Wang S., Zhu L., Dong L.Y., Zhang Z.T., Hao B., Yang F., Liu W.B., Deng Y. (2017). Meteorological factors affect the epidemiology of hemorrhagic fever with renal syndrome via altering the breeding and hantavirus-carrying states of rodents and mites: A 9 years’ longitudinal study. Emerg. Microbes Infect..

[16] Zhang Y., Zhu J., Tao K., Wu G., Guo H., Wang J., Zhang J., Xing A. (2002). Proliferation and location of Hantaan virus in gamasid mites and chigger mites, a molecular biological study. Natl. Med. J. China.

[17] Renz A., Wenk P. (1981). Intracellular development of the cotton-rat filaria *Litomosoides carinii* in the vector mite *Ornithonyssus bacoti*. T. Roy. Soc. Trop. Med. H..

[18] Xi Y.F., Xu J.J., Ren Y.F., Yuan Y.Z., Tao G.Y., Zhao M.L., Xu Y.X. (1979). Drug screening with the cotton-rat model of filariasis. Acta Pharm. Sin..

[19] Ming Q.Z., Shi Z.T. (2006). The tentative inquiry on the formation time in the region of three parallel rivers. Yunnan Geog. Environ. Res..

[20] Zhang R.Z. (2002). Geological events and mammalian distribution in China. Acta Zool. Sin..

[21] Wu S.H., Dai E.F., He D.M. (2005). Major Research Perspectives on Environmental and Developmental Issues for the Longitudinal Range-Gorge Region (LRGR) in Southwestern China. Prog. Geog..

[22] Ma C.L., Robert K M., Chen W.Y., Zhou Z.K. (2007). Plant diversity and priority conservation areas of Northwestern Yunnan, China. Biodivers. Conserv..

[23] Moseley R.K. (2006). Historical Landscape Change in Northwestern Yunnan, China. Mt. Res. Dev..

[24] Sherman R., Mullen R., Li H., Fang Z., Yi W. (2008). Spatial patterns of plant diversity and communities in alpine ecosystems of the Hendguan Mountains, Northwest Yunnan, China. J. Plant Ecol..

[25] Zhang R.Z., Zheng D., Yang Q.Y., Liu Y.H. (1997). The Series of the Scientific Expedition to Hengduan Mountains, Qinghai-Xizang Plateau. Physical Geography of Hengduan Mountains.

[26] Lin S., Wu R., Hua C., Ma J., Wang W., Yang F., Wang J. (2016). Identifying local-scale wilderness for on-ground conservation actions within a global biodiversity hotspot. Sci. Rep..

[27] Diao Y., Wang J., Yang F., Wu W., Zhou J., Wu R. (2021). Identifying optimized on-the-ground priority areas for species conservation in a global biodiversity hotspot. J. Environ. Manag..

[28] Sherman R., Mullen R. (2007). Alpine Ecosystems of Northwest Yunnan, China: An Initial Assessment for Conservation. J. Mountain Sci..

[29] Dahmana H., Granjon L., Diagne C., Davoust B., Fenollar F., Mediannikov O. (2020). Rodents as Hosts of Pathogens and Related Zoonotic Disease Risk. Pathogens.

[30] Gong Z.D., Wu H.Y., Duan X.D., Feng X.G., Zhang Y.Z., Liu Q. (2001). The species diversity and distribution trends of small mammals in Hengduan Mountains Yunnan. Biodivers. Sci..

[31] Quan S.Y., Yue R.P., Zhang L.Y., Lian H.Y., Zang Y.H., Bian C.L., Li D., Ju J.K., Gong Z.D. (2010). The composition and spatial distribution of small mammals in the Hengduan Mountains of Yunnan, China. Chin. J. Vector Biol. Control.

[32] Gao G., Wang B., He C.X., Luo X. (2017). Biodiversity of birds and mammals in alpine habitat of Mt. Gaoligong, Lushui County, Yunnan. Biodivers. Sci..

[33] Chen Z.Z., Li X.Y., Song W.Y., Li Q., Onditi K., Khanal L., Jiang X.L. (2020). Small mammal species richness and turnover along elevational gradient in Yulong Mountain, Yunnan, Southwest China. Ecol. Evol..

[34] Wilson D.E., Lacher T.E., Mittermeier R.A. (2017). Handbook of the Mammals of the World, vol 7, Rodents II.

[35] Smith A.T., Xie Y., Hoffmann R.S., Lunde D., MacKinnon J., Wilson D.E., Wozencraft W.C. (2008). A Guide to the Mammals of China.

[36] Huang W.J., Chen Y.X., Wen Y.X. (1995). Glires of China (Zhong Guo Nie Chi Lei).

[37] Pan Z.W., Deng G.F. (1980). Economic Insect Fauna of China. Fasc 17, Acari: Gamasina.

[38] Evans G.O., Till W.M. (1979). Mesostigmatic mites of Britain and Ireland (Chelicerata: Acari-Parasitiformes): An introduction to their external morphology and classification. Trans. Zool. Soc. Lond..

[39] Radovsky F.J. (1967). The Macronyssidae and Laelapidae (Acarina: Mesostigmata) Parasitic on Bats.

[40] Chen Y.L., Guo X.G., Ren T.G., Zhang L., Fan R., Zhao C.F., Zhang Z.W., Mao K.Y., Huang X.B., Qian T.J. (2022). Infestation and distribution of chigger mites on Chevrieri’s field mouse (*Apodemus chevrieri*) in Southwest China. Int. J. Parasitol. Parasites Wildl..

[41] Peng P.Y., Guo X.G., Ren T.G., Song W.Y., Dong W.G., Fan R. (2016). Species diversity of ectoparasitic chigger mites (Acari: Prostigmata) on small mammals in Yunnan Province, China. Parasitol. Res..

[42] Legendre P. (2007). Studying beta diversity: Ecological variation partitioning by multiple regression and canonical analysis. J. Plant Ecol..

[43] Legendre P. (2008). Studying beta diversity: Ecological variation partitioning by multiple regression and canonical analysis. J. Plant Ecol..

[44] Guo X.G., Dong W.G., Men X.Y., Qian T.J., Wu D., Ren T.G., Qin F., Song W.Y., Yang Z.H., Fletcher Q.E. (2016). Species Abundance Distribution of Ectoparasites on Norway Rats (*Rattus norvegicus*) from a Localized Area in Southwest China. J. Arthropod Borne Dis..

[45] Peng P.Y., Guo X.G., Jin D.C., Dong W.G., Qian T.J., Qin F., Yang Z.H. (2017). Species abundance distribution and ecological niches of chigger mites on small mammals in Yunnan province, southwest China. Biologia.

[46] Baltanás A. (1992). On the Use of Some Methods for the Estimation of Species Richness. Oikos.

[47] Guo X.G., Speakman J.R., Dong W.G., Men X.Y., Qian T.J., Wu D., Qin F., Song W.Y. (2013). Ectoparasitic insects and mites on Yunnan red-backed voles (*Eothenomys miletus*) from a localized area in southwest China. Parasitol. Res..

[48] Chen Z., Yang X.J., Liu J.Z. (2006). Research report of medical ticks and mites in Hebei province. J. Med. Pest Control.

[49] Ji H.Q., Feng S.Q., Liu N., He Y.M., Li H., Zhu B., Du J., Zhou C.B. (2012). Species and geographical distribution of fleas and gamasid mites on the rat-shape animals in Chongqing city. Chin. J. Hyg. Insectic. Equip..

[50] Lu M.G., Jiang Q.L., Gong Z.Y., Ni Q.X., Ma L.M. (2017). A list of Gamasid mites (Acari: Gamasina) in Zhejiang province. Chin. J. Vector Biol. Control.

[51] Tao J.W., Liu Y.R., Yang Z.Q. (2005). Analysis on Fauna of Gamasid Mites in Hubei Province, China. Chin. J. Vector Biol. Control.

[52] Zhou G.Z., Wang Z., Huang W.C., Li P., Yin G.Q., Wen Y., Cheng X.H., Xue J. (2013). Survey of gamasid mite species and evaluation of gamasid mite control efficacy in military camps in Shandong province, China. Chin. J. Vector Biol. Control.

[53] Zhou S.H., Deng Y.Q., Li S.Y., Wang L.L. (2012). Supplementary records of Dermanyssoid mites (Acari: Parasitiformes) in Fujian province. Chin. J. Vector Biol. Control.

[54] Mašán P. (2017). A revision of the family Ameroseiidae (Acari, Mesostigmata), with some data on Slovak fauna. ZooKeys.

[55] Vinarski M.V., Korallo-Vinarskaya N.P. (2020). An annotated catalogue of the gamasid mites associated with small mammals in Asiatic Russia. The family Hirstionyssidae (Acari: Mesostigmata: Gamasina). Zootaxa.

[56] Salmane I., Heldt S. (2001). Predatory soil mites (Acari, Mesostigmata, Gamasina) from the Western Baltic Coast of Latvia. Acarologia.

[57] He J., Lin S., Li J., Yu J., Jiang H. (2020). Evolutionary history of zoogeographical regions surrounding the Tibetan Plateau. Commun. Biol..

[58] Kai H., Jiang X. (2014). Sky islands of southwest China. I: An overview of phylogeographic patterns. Chin. Sci. Bull..

[59] Wang Y., Si Tu Q., Li Z.H. (2003). Relationship Between Regional Geographic Environment Characteristics and Soil and Water Loss of Northwest Yunnan Province. Bull. Soil Water Conserv..

[60] Cavarzere V., Roper J.J., Marchi V., Silveira L.F. (2021). Geographical drivers of altitudinal diversity of birds in the Atlantic Forest. Biologia.

[61] Ghimire A., Rokaya M.B., Timsina B., Bílá K., Kindlmann P. (2021). Diversity of birds recorded at different altitudes in central Nepal Himalayas. Ecol. Indic..

[62] Heaney L.R. (2001). Small mammal diversity along elevational gradients in the Philippines: An assessment of patterns and hypotheses. Global Ecol. Biogeogr..

[63] Koh C.N., Lee P.F., Lin R.S. (2010). Bird species richness patterns of northern Taiwan: Primary productivity, human population density, and habitat heterogeneity. Divers. Distrib..

[64] Gao J., Zhang X., Luo Z.F., Lan J.J., Liu Y.H. (2018). Elevational diversity gradients across seed plant taxonomic levels in the Lancang River Nature Reserve: Role of temperature, water and the mid-domain effect. J. For. Res..

[65] Christy M.M. (2010). Global analysis of reptile elevational diversity. Global Ecol. Biogeogr..

[66] Xiang R., Guo X.G., Ren T.G., Zhao C.F., Fan R., Zhang Z.W., Mao K.Y., Peng P.Y., Huang X.B., Qian T.J. (2021). Infestation and distribution of mites on the Yunnan red-backed vole (*Eothenomys miletus*) in Yunnan Province of southwest China between 2001 and 2015. Biologia.

[67] Li B., Guo X.G., Zhao C.F., Zhang Z.W., Fan R., Peng P.Y., Song W.Y., Ren T.G., Zhang L., Qian T.J. (2022). Infestation of chigger mites on Chinese mole shrew, *Anourosorex squamipes*, in Southwest China and ecological analysis. Parasite.

[68] Eslami A., Yousefi A., Dowling A.P.G. (2018). Prevalence of ectoparasites in black rat (*Rattus rattus*) from Mangrove forests of Qeshm Island, Iran. Comp. Clin. Path..

[69] Peng P.Y., Guo X.G., Song W.Y., Hou P., Zou Y.J., Fan R. (2016). Ectoparasitic chigger mites on large oriental vole (*Eothenomys miletus*) across southwest, China. Parasitol. Res..

[70] Fecchio A., Martins T.F., Bell J.A., De La Torre G.M., Pinho J.B., Weckstein J.D., Tkach V.V., Labruna M.B., Dias R.I. (2020). Low host specificity and lack of parasite avoidance by immature ticks in Brazilian birds. Parasitol. Res..

[71] Ding F., Guo X.G., Song W.Y., Fan R., Zhao C.F., Mao K.Y., Zhang Z.W., Peng P.Y., Lin H., Dong W.G. (2021). Infestation and distribution of chigger mites on Brown rat (*Rattus norvegicus*) in Yunnan Province, Southwest China. Trop. Biomed..

[72] Bhuyan P.J., Nath A.J. (2015). Record of Tropical Rat Mite, *Ornithonyssus bacoti* (Acari: Mesostigmata: Macronyssidae) from Domestic and Peridomestic Rodents (*Rattus rattus*) in Nilgiris, Tamil Nadu, India. J. Arthropod Borne Dis..

[73] Heukelbach J., Feldmeier H. (2005). Ectoparasitic Infestations. Curr. Infect. Dis. Rep..

[74] McGill B.J., Etienne R.S., Gray J.S., Alonso D., Anderson M.J., Benecha H.K., Dornelas M., Enquist B.J., Green J.L., He F. (2007). Species abundance distributions: Moving beyond single prediction theories to integration within an ecological framework. Ecol. Lett..

[75] Guo X.G., Qian T.J., Meng X.Y., Dong W.G., Shi W.X., Wu D. (2006). Preliminary ananlysis of chigger communities associated with house rats (*Rattus flavipectus*) from six counties in Yunnan, China. Syst. Appl. Acarol..

[76] Engen S. (2007). Heterogeneous communities with lognormal species abundance distribution: Species–area curves and sustainability. J. Theor. Biol..

[77] Ding F., Jiang W.L., Guo X.G., Fan R., Zhao C.F., Zhang Z.W., Mao K.Y., Xiang R. (2021). Infestation and Related Ecology of Chigger Mites on the Asian House Rat (*Rattus tanezumi*) in Yunnan Province, Southwest China. Korean J. Parasitol..

[78] Matthews T.J., Whittaker R.J. (2015). On the species abundance distribution in applied ecology and biodiversity management. J. Appl. Ecol..

